# Clinicopathological features and prognostic outcomes of molecularly defined entities in the new edition of the WHO classification of sinonasal carcinoma

**DOI:** 10.3389/fonc.2023.1117865

**Published:** 2023-03-01

**Authors:** Huy Gia Vuong, Thoa Le, Trang T.B. Le, Hieu Trong Le, Edward T. El-Rassi, Kibwei A. McKinney, Ian F. Dunn

**Affiliations:** ^1^ Department of Pathology, University of Iowa Hospitals and Clinics, Iowa City, IA, United States; ^2^ Faculty of Medicine, University of Medicine and Pharmacy at Ho Chi Minh City, Ho Chi Minh City, Vietnam; ^3^ Department of Medicine, University of Medicine and Pharmacy at Ho Chi Minh City, Ho Chi Minh City, Vietnam; ^4^ Department of Otolaryngology, University of Oklahoma Health Sciences Center, Oklahoma City, OK, United States; ^5^ Department of Neurosurgery, University of Oklahoma Health Sciences Center, Oklahoma City, OK, United States

**Keywords:** sinonasal carcinoma, sinonasal undifferentiated carcinoma, *SMARCB1*, *SMARCA4*, IDH2, nut

## Abstract

**Introduction:**

We investigated the clinicopathological features and prognoses of the new molecularly defined entities in latest edition of the World Health Organization (WHO) classification of sinonasal carcinoma (SNC)

**Methods:**

Integrated data were combined into an individual patient data (IPD) meta-analysis.

**Results:**

We included 61 studies with 278 SNCs including 25 *IDH2-*mutant, 41 NUT carcinoma, 187 SWI/SNF loss, and 25 triple negative SNCs (without IDH2 mutation, *NUTM1* rearrangement, and SWI/SNF inactivation) for analyses. Compared to other molecular groups, NUT carcinoma was associated with a younger age at presentation and an inferior disease-specific survival. Among SNCs with SWI/SNF inactivation, *SMARCB1-*deficient tumors presented later in life and were associated with a higher rate of radiotherapy administration. *SMARCA4-*deficiency was mostly found in teratocarcinosarcoma while *SMARCB1-*deficient tumors were associated with undifferentiated carcinoma and non-keratinizing squamous cell carcinoma.

**Conclusion:**

Our study facilitates our current understanding of this developing molecular-defined spectrum of tumors and their prognoses.

## Introduction

Sinonasal carcinomas (SNC) are rare malignancies and are usually associated with poor outcomes. In the previous editions of the World Health Organization (WHO) Classification, sinonasal malignancies were mainly classified as conventional squamous cell carcinoma (SCC), non-keratinizing SCC, intestinal-type adenocarcinoma (ITAC), non-ITAC, neuroendocrine carcinoma (NEC), poorly differentiated carcinoma (PDCA), sinonasal undifferentiated carcinoma (SNUC), and other rare subtypes (1). The 2022 5^th^ edition of the WHO Classification of the Head and Neck has made significant classification revisions, with newly added molecular groups for SNC as compared to previous versions (1).

SWI/SNF complex-deficient carcinomas, defined by loss of one of the SWI/SNF complex genes, include two major subtypes: *SMARCB1*- and *SMARCA4*-deficient sinonasal carcinoma (2–4). Most of these cases were previously misdiagnosed as PDCA, SNUC, NEC, or teratocarcinosarcoma (TCS). Mutations in *IDH2* have also been recently described in a subset of PDCA and SNUC (5, 6). Tumors with these mutations are generally associated with better outcomes relative to those without *IDH2* mutations (7, 8); however, results to the contrary have also been reported (9). Because of the rarity of these new entities, we lack a detailed understanding of the clinicopathological features and prognoses between them. This meta-analysis aimed to investigate the clinicopathological characteristics and survival patterns of SWI/SNF-deficient and *IDH2-*mutant tumors in comparison to the previously described NUT midline carcinoma of the sinonasal tract.

## Materials and methods

### Literature search and search term

Relevant articles were found by searching three electronic databases including PubMed, Web of Science, and Scopus from their inception to September 2022. We used the following search terms: (sinonasal OR nasal OR paranasal) AND (carcinoma OR cancer) AND (SMARCB1 OR SMARCB-1 OR SMARCB 1 OR INI1 OR INI 1 OR INI-1 OR SMARCA4 OR SMARCA-4 OR SMARCA 4 OR BRG1 OR BRG-1 OR BRG 1 OR SWI/SNF OR NUT OR isocitrate OR IDH1/2 OR IDH2). We carefully reviewed the reference list of potential articles to avoid missing important data. This study protocol strictly followed the recommendations of Preferred Reporting Items for Systematic Review and Meta-analysis (PRISMA) statement (10).

### Selection criteria, abstract/full text screening

For abstract screening, two independent teams (HGV, TL, TTBL, and HTL) reviewed the titles and abstracts of included articles. Studies were included if they are observational studies and report individual patient data (IPD) of *SMARCB1-*deficient, *SMARCA4-*deficient, NUT midline, and *IDH2-*mutant carcinoma of the sinonasal tract. We excluded studies if they are (i) reviews, (ii) conference abstracts or conference papers, (iii) books, (iv) without IPD, and (iv) duplicated data.

Following this step, two independent teams read all full texts of potential studies and extracted data into a standardized worksheet. The following data were collected: author names, institution, city, country, publication year, number of patients, age, gender, clinicopathological information (e.g., tumor location, largest diameter, tumor extension, nodal/distant metastases, TNM stage, original histological diagnosis, number of mitoses per 10 high-power filed, Ki67 index), treatments administered, progression-free survival (PFS), and disease-specific survival (DSS).

### Statistical analysis

We divided data into four main groups: *SWI/SNF* loss, NUT carcinoma, *IDH2*-mutant, and those without *SWI/INF* deficiency, *NUTM1* fusion, and *IDH2* mutation (triple negative group). We excluded cases that were absent the *NUTM1* rearrangement and *SWI/SNF* loss but missing information on *IDH2* status. For *SWI/INF-*deficient tumors, we also compared the *SMARCB1-*deficient versus *SMARCA4*-deficient carcinomas. We used Chi-squared and Fisher’s exact test for comparison of categorical variables while t-test, Wilcoxon rank sum test, or analysis of variance (ANOVA) were utilized for continuous covariates, if applicable. The R program (The R Foundation, Vienna, Austria) was used for statistical analyses.

## Results

**Figure 1:**
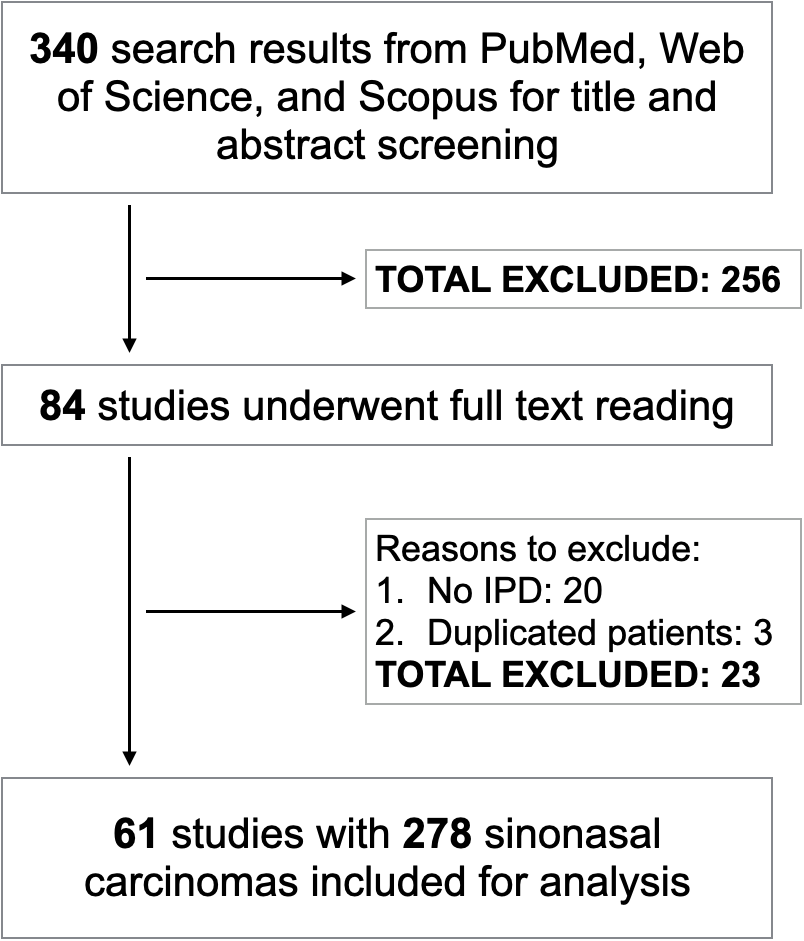
Study flowchart.

After merging search results from three electronic databases and removing the duplicates, we had 340 studies for title and abstract screening. Following this step, 84 articles were selected for full-text reading. Sixty-one of them met inclusion criteria corresponding to 278 SNCs which were included for analysis (2–5, 9, 11–66) (**Figure 1**). There were 25 *IDH2-*mutant, 41 NUT carcinoma, 187 SWI/SNF loss, and 25 triple negative SNCs. Among NUT carcinoma, *BRD4:NUTM1* was the most common variant and only one case harbored *BRD3:NUTM1* rearrangement. The R172 variant was the most predominant *IDH2-*mutant genotype. Regarding SNCs with inactivation of one of the SWI/SNF complex genes, *SMARCB1-*deficient carcinoma was the most frequent subtype followed by *SMARCA4-*deficient tumors. Loss of *SMARCA1, SMARCA5*, and *SMARCE1* were also found in one SNC case each. *IDH2* mutations, *NUTM1* rearrangement, and inactivation of SWI/SNF complex were mutually exclusive with each other.

### Clinicopathological features and treatment patterns of molecular groups of SNCs

**Table 1** describes the clinicopathological and therapeutic parameters of different molecular groups of SNCs. Compared to *IDH2-*mutant, SWI/SNF loss, and triple negative groups, NUT carcinoma presented a significantly younger age (p < 0.001). Most *IDH2-*mutant SNCs were originally diagnosed as SNUC whereas the diagnosis of NUT carcinoma is usually more straightforward. SWI/SNF-loss SNCs were commonly misdiagnosed as SNUC, SCC, or TCS. Lymph node metastases were generally uncommon in SNCs whereas distant metastases were more frequently observed among all molecular groups.

**Table 1:** Clinicopathological characteristics and treatment patterns of different molecular subgroups of SNCs.

Parameters	*IDH2*-mut	NUT carcinoma	SWI/SNF loss	Triple negative	*p*-value
	(N=25)	(N=41)	(N=187)	(N=25)	
Age					< 0.001
Mean (SD)	57.3 (13.5)	40.7 (17.7)	50.8 (17.5)	57.4 (14.3)	
Median [Min, Max]	53.5 [39.0, 83.0]	42.0 [0.750, 77.0]	51.0 [11.0, 95.0]	54.0 [30.0, 81.0]	
Gender					0.194
Female	5 (31.3%)	20 (48.8%)	59 (33.3%)	10 (47.6%)	
Male	11 (68.8%)	21 (51.2%)	118 (66.7%)	11 (52.4%)	
**Nodal metastasis**	3 (21.4%)	6 (19.4%)	20 (16.0%)	2 (13.3%)	0.884
**Distant metastasis**	4 (28.6%)	10 (32.3%)	45 (35.7%)	7 (46.7%)	0.754
Original diagnoses					< 0.001
Adenocarcinoma	1 (4.0%)	0 (0%)	7 (4.3%)	3 (12.0%)	
NEC	3 (12.0%)	0 (0%)	13 (7.9%)	3 (12.0%)	
PDCA	1 (4.00%)	10 (28.6%)	12 (7.3%)	7 (28.0%)	
SNUC	20 (80.0%)	3 (8.6%)	46 (28.0%)	8 (32.0%)	
Teratocarcinosarcoma	0 (0%)	0 (0%)	23 (14.0%)	4 (16.0%)	
* NUT* carcinoma	0 (0%)	14 (40.0%)	0 (0%)	0 (0%)	
SCC	0 (0%)	7 (20.0%)	30 (18.3%)	0 (0%)	
* SMARCB1*-deficient	0 (0%)	0 (0%)	26 (15.9%)	0 (0%)	
Other	0 (0%)	1 (2.8%)	7 (4.3%)	0 (0%)	
Resection					0.634
Biopsy	3 (25.0%)	11 (33.3%)	34 (23.0%)	4 (26.7%)	
Resection	9 (75.0%)	22 (66.7%)	114 (77.0%)	11 (73.3%)	
**Radiotherapy**	13 (92.9%)	26 (89.7%)	102 (70.8%)	14 (93.3%)	**0.020**
**Chemotherapy**	11 (78.6%)	20 (69.0%)	94 (65.3%)	11 (73.3%)	0.788
**Progression/Recurrence**	7 (53.8%)	12 (54.5%)	64 (59.8%)	9 (60.0%)	0.951

NEC, neuroendocrine carcinoma; PDCA, poorly differentiated carcinoma; SCC, squamous cell carcinoma; SNUC, sinonasal undifferentiated carcinoma. Bold values indicate statistically significant result.

Regarding treatments, SWI/SNF-loss SNCs were less likely to receive radiotherapy as compared to other groups (p = 0.020). The rate of nodal metastasis, distant metastasis, surgical resection, and chemotherapy administration were statistically comparable between the four groups.

We also sought to investigate the similarities and differences between *SMARCA4-*deficient versus *SMARCB1-*deficient SNCs (**Table 2**). *SMARCA4-*deficient SNCs presented at a significantly younger age compared to *SMARCB1-*deficient (median, 42.0 versus 53.0). A subset of *SMARCA4-*deficient SNCs had overlapping histopathological findings with TCS whereas *SMARCB1-*deficient were distributed in more diverse histological diagnoses. Radiotherapy administration was more commonly used for *SMARCB1-*deficient tumors.

**Table 2:** Clinicopathological features and treatment patterns of *SMARCA4-*deficient versus *SMARCB1-*deficient SNCs.

Parameters	*SMARCA4*-def	*SMARCB1*-def	*p*-value
	(N=45)	(N=139)	
Age			0.001
Mean (SD)	43.1 (14.8)	53.2 (17.7)	
Median [Min, Max]	42.0 [18.0, 70.0]	53.0 [11.0, 95.0]	
Gender			0.901
Female	14 (32.6%)	45 (33.6%)	
Male	29 (67.4%)	89 (66.4%)	
**Nodal metastasis**	1 (5.0%)	19 (18.6%)	0.191
**Distant metastasis**	7 (35.0%)	38 (36.9%)	0.872
Original diagnoses			< 0.001
Adenocarcinoma	0 (0%)	7 (5.8%)	
NEC	12 (29.3%)	1 (0.9%)	
PDCA	2 (4.9%)	10 (8.3%)	
SCC	1 (2.4%)	29 (24.2%)	
SNUC	3 (7.3%)	40 (33.3%)	
Teratocarcinosarcoma	23 (56.1%)	0 (0%)	
* SMARCB1*-deficient	0 (0%)	26 (21.7%)	
Other	0 (0%)	7 (5.8%)	
Resection			0.411
Biopsy	10 (28.6%)	24 (21.8%)	
Resection	25 (71.4%)	86 (78.2%)	
**Radiotherapy**	14 (43.8%)	85 (78.0%)	< 0.001
**Chemotherapy**	18 (56.3%)	73 (67.0%)	0.265

NEC, neuroendocrine carcinoma; PDCA, poorly differentiated carcinoma; SCC, squamous cell carcinoma; SNUC, sinonasal undifferentiated carcinoma.

### Metastatic patterns of SNCs

Bone and lung were the two most common metastatic sites for SNCs. We found significant different metastatic patterns of *IDH2-*mutant SNCs as compared to other groups. No *IDH2-*mutant SNCs metastasized to lung and most of these tumors had a metastatic preference to liver and other rare organs (e.g., adrenal glands, mediastinum) (**Table 3**).

**Table 3:** Metastatic patterns of SNCs.

Metastatic sites	*IDH2*-mutant	NUT carcinoma	SWI/SNF loss	Triple negative	Overall	*p*-value
Lung	0 (0%)	5 (23.8%)	19 (19.2%)	2 (13.3%)	27 (18.1%)	0.297
Bone	2 (15.4%)	5 (23.8%)	18 (18.2%)	5 (33.3%)	30 (20.1%)	0.501
Brain	0 (0%)	1 (4.8%)	9 (9.1%)	0 (0%)	11 (7.4%)	0.710
Liver	3 (23.1%)	4 (19.0%)	4 (4.0%)	2 (13.3%)	14 (9.4%)	0.012
Soft tissue	0 (0%)	1 (4.8%)	2 (2.0%)	0 (0%)	3 (2.01%)	0.704
Others	2 (15.4%)	3 (14.3%)	2 (2.0%)	1 (6.7%)	8 (5.4%)	0.019

### Prognoses of molecular groups of SNCs

**Figure 2:**
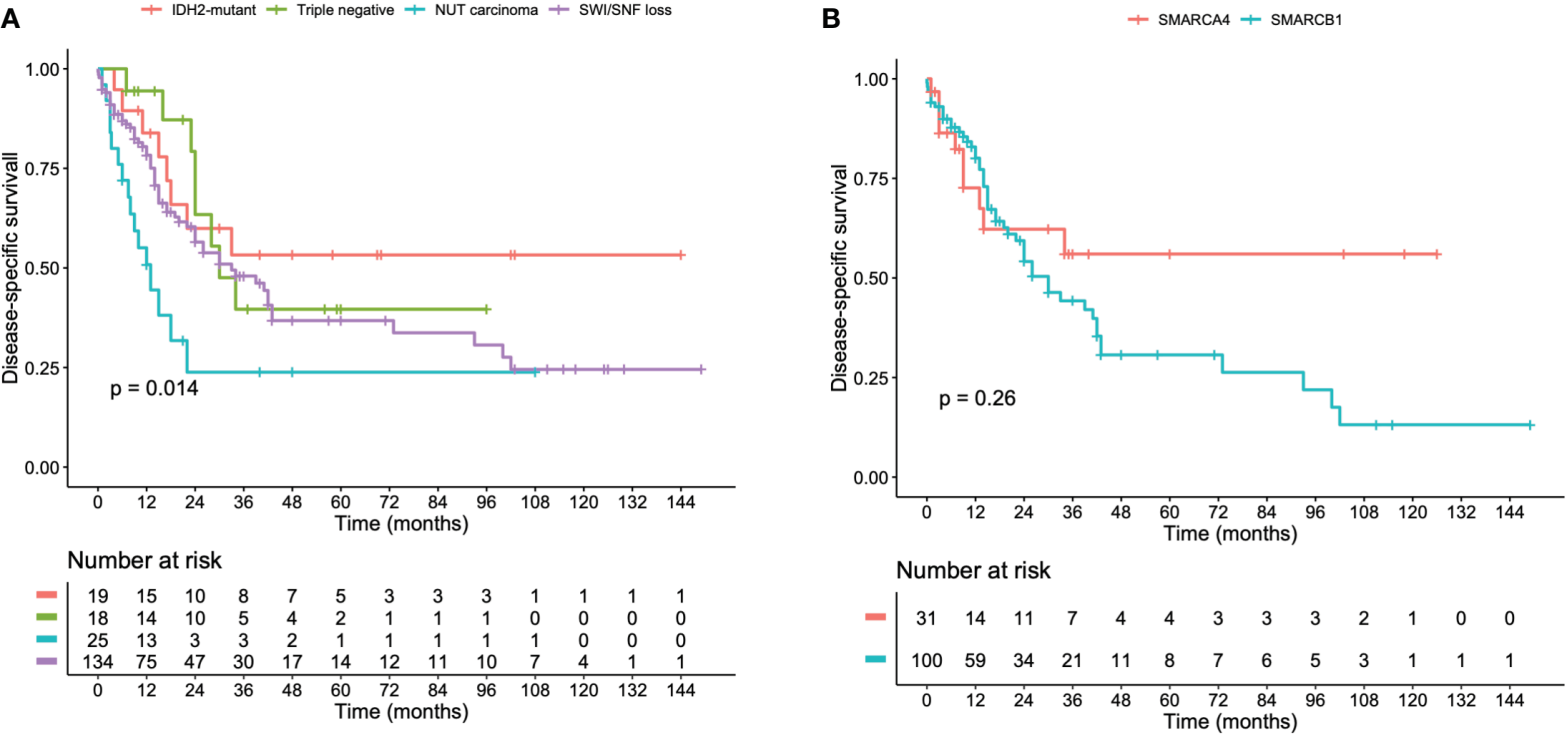
Kaplan-Meier curves illustrating the DSS of **(A)** *IDH2-*mutant, NUT midline, SWI/SNF-loss, and triple negative sinonasal carcinomas. **(B)** *SMARCA4-*deficient and *SMARCB1-*deficient sinonasal carcinomas.

SNCs were associated with high-risk for local relapse and tumor progression during follow-up. We could not calculate and compare the PFS between the molecular groups due to high rate of missing data. Kaplan-Meier analyses demonstrated that *IDH2-*mutant and triple negative SNCs have a more favorable DSS compared to NUT carcinoma (p = 0.014) (**Figure 2A**). The DSS was not statistically different between *SMARCA4-*deficient versus *SMARCB1-* deficient SNCs (**Figure 2B**). In a multivariate Cox regression model, NUT carcinoma and no radiotherapy administration were prognostic indicators for poor prognosis (**Table 4**).

**Table 4:** Multivariate Cox regression analysis for DSS of SNCs.

Parameter		HR (95% CI)	p-value
Age	Per year increase	1.003 (0.989-1.018)	0.671
Gender	Female	Reference	
	Male	1.006 (0.611-1.656)	0.981
Molecular subgroups	Triple negative	Reference	
	*IDH2*-mut	0.624 (0.178-2.193)	0.462
	NUT carcinoma	2.908 (1.130-7.483)	0.027
	SWI/SNF loss	1.210 (0.541-2.707)	0.643
Extent of surgery	Biopsy	Reference	
	Resection	0.868 (0.437-1.726)	0.687
Radiotherapy	No	Reference	
	Yes	0.328 (0.173-0.621)	< 0.001
Chemotherapy	No	Reference	
	Yes	0.955 (0.529-1.721)	0.877

## Discussion

In recent years, new molecular profiles of SNCs have been further clarified and novel molecular groups have been incorporated into the latest WHO classification of SNCs (1–6). Prior to the molecular era, most *IDH2-*mutant, NUT midline, and SWI/SNF-deficient SNCs were categorized as SNUC, PDCA, TCS, or NEC (2, 3, 5, 33). In the latest WHO classification of head and neck tumors, *NUT* midline, *SMARCA4*-deficient, and *SMARCB1-*deficient SNCs have been recognized as separate entities. Given their distinct clinicopathological features and prognoses as compared to *IDH2-*wild type tumors (8), *IDH2*-mutant SNCs may nevertheless be regarded as a distinct molecular group in future WHO editions. Because of the rarity of SNCs, most data were presented as case reports or small- to medium-sized case series. The clinicopathological features and prognostic outcomes of new molecular groups of SNCs have been described. However, it is still controversial as to how these tumors are different from each other and in how clinicians can better assess patient outcomes. In this study, we integrated IPD of published studies into a meta-analysis to improve the statistical implication compared to cohort studies with limited sample size.

Our results showed that these tumors were uniformly high-grade and distributed in diverse histopathological spectrums with SNUC and PDCA being the most common variants. All molecular groups of SNCs had a relatively considerable risk for tumor metastases to distant organs with bone and lung being the most common sites. We found that *IDH2-*mutant SNCs were most likely to metastasize to liver and other rare organs compared to other groups. Like prior studies, our meta-analysis demonstrated improved survival of *IDH2-*mutant SNCs as compared to those without these mutations (5, 6, 8). The prognostic implication of *IDH1/2* mutations in gliomas, chondrosarcoma, and cholangiocarcinoma have similarly been established (67–70). The discovery of *IDH2* mutation in SNCs provides a promising opportunity for targeted therapy with IDH inhibitors. Most *IDH2-*mutant SNCs occur in codon 172 and can be diagnosed by immunohistochemistry assay which is an accessible, rapid, and inexpensive method (8, 31).

This meta-analysis also highlighted that SNCs usually present at older age except for NUT carcinoma, which is more commonly seen in young adults and pediatric patients. NUT carcinoma is exceedingly rare, typically occurs in the midline structures, and histopathologically resembles PDCA. This tumor is characterized by a chromosomal rearrangement involving *NUTM1* gene (32). The availability of NUT immunohistochemistry antibody has improved the accuracy of NUT midline carcinoma diagnosis and differentiated them from other PDCA. NUT midline carcinoma is associated with high rates of mortality (71) and our study further confirmed the uniformly poor prognosis of these tumors compared to other genetic groups of SNCs.

The most common genetically defined group of SNCs involves the SWI/SNF complex genes with loss of *SMARCB1* and *SMARCA4* being the most common variants. It is still poorly understood regarding how these subtypes are different from each other. *SMARCB1-*loss SNC is associated with rhabdoid differentiation in SNUC (2), which is an important diagnostic parameter to differentiate them from other PDCA. On the other hand, recurrent loss of SMARCA4 is commonly observed in TCS (3) and SNCs with neuroendocrine differentiation (4). Our analyses further confirmed these histopathological associations. We also found that *SMARCB1-*deficient SNCs occur at a significantly older age and more likely to have radiotherapy administration in comparison to *SMARCA4-*deficient tumors. From our analysis, the DSS of these two new SNC entities were comparable. With the use of immunohistochemistry, it is easier to recognize these two rare entities and separate them from other sinonasal PDCAs.

This study is the first meta-analysis comparing the new molecular groups of SNCs in the new edition of WHO classification. It helps summarize and facilitate our current understanding about the clinicopathological behaviors and prognoses of these aggressive tumors. However, there are certain limitations. First, all included studies are retrospective cohort studies or case reports/series leading to inevitable selection bias. Next, we could not include other recently described molecular entities such as *DEK::AFF2-*rearranged non-keratinizing SCC *and TP53-*mutant ITAC due to limited data. In addition, we could not compare the effectiveness of treatment modalities in each molecularly defined SNC subgroup due to missing data. Finally, we could not assess PFS, an important prognostic value due to missing data in most included studies. Future large multicenter prospective studies are essential to validate the results of this study.

In summary, the evolution of molecular pathology alongside standard immunohistochemistry enables us to recognize and accurately diagnose novel molecular entities of SNCs. These tumors have distinct clinicopathological profiles and prognoses and should be distinguished from other SNCs to better understand their unique natural histories and treatment implications.

## Author contributions

HV: conceptualization, data curation, formal analysis, investigation, methodology, project administration, software, validation, writing original, review, and editing. TL: data curation, formal analysis, investigation, methodology, review, and editing. TTBL: data curation, formal analysis, investigation, methodology, review, and editing. HL: data curation, formal analysis, investigation, methodology, review, and editing. EE-R: data curation, formal analysis, investigation, methodology, review, and editing. KM: data curation, formal analysis, investigation, methodology, review, and editing. ID: conceptualization, project administration, validation, review, editing, and supervisions. All authors contributed to the article and approved the submitted version.
